# Anterior-Segment Optical Coherence Tomography-Based Evaluation of the Journey of a Bleb in the Early Postoperative Period

**DOI:** 10.7759/cureus.63512

**Published:** 2024-06-30

**Authors:** Tanvi S Choudhary, Reema M Raval, Kintu S Shah, Sakshi M Gajwani, Radha J Mehta, Megha C Patel

**Affiliations:** 1 Glaucoma and Squint Clinic, Shri Chimanlal Harilal (CH) Nagri Municipal Eye Hospital, Ahmedabad, IND; 2 Department of Psychiatry, Gujarat Medical Education and Research Society (GMERS) Medical College and Hospital, Navsari, IND; 3 Department of Pedodontics and Preventive Dentistry, Karnavati School of Dentistry, Karnavati University, Gandhinagar, IND

**Keywords:** tomography-based evaluation, bleb wall height, intraocular pressure measurement, bleb wall reflectivity, bleb wall thickness, optical coherence tomography (oct), bleb morphology, as-oct, trabeculectomy bleb

## Abstract

Introduction

In the early postoperative period following trabeculectomy, monitoring the journey of bleb formation is crucial for assessing surgical success. Anterior-segment optical coherence tomography (AS-OCT) emerges as a powerful tool in this pursuit, offering high-resolution imaging of bleb morphology and dynamics. This study aims to evaluate the internal structure of blebs through their maturation phases using AS-OCT.

Methods

Fifty-five eyes undergoing trabeculectomy were enrolled in a prospective observational study. Serial AS-OCT examinations were done on day 1, week 1, week 3, and week 6 postoperatively; bleb parameters were calculated and correlated with intraocular pressure (IOP).

Results

IOP control was seen in 45 eyes six months of post-trabeculectomy. Multiform bleb wall reflectivity (BWR) statistically correlates with the success of trabeculectomy. Blebs were successful if BWR showed no change from day 1 to week 6. BWR remained the same on all follow-ups if week 1 bleb wall thickness (BWT) was less than 129.5 microns with 82.6% sensitivity and 83.3% specificity. The cumulative hazard of change in BWR is estimated to be approximately 5.6%, 15.7%, and 17.9% at week 1, week 3, and week 6 follow-ups, respectively.

Conclusions

Successful blebs showed consistent BWR from day 1 to week 6 of follow-up. Serial AS-OCT examination for changes in BWR in early stages can be done to predict the fate of bleb. The maximum change in BWR occurs between the week 1 and week 3 follow-up periods requiring close follow-up.

## Introduction

Trabeculectomy is a time-tested procedure for treating glaucoma. The outcome of this surgery depends on forming a functioning filtration bleb that enables the egress of aqueous from the eye [1]. Bleb morphology is an essential clinical parameter that decides the success of trabeculectomy.

Previous studies have utilized various methodologies to grade and categorize blebs, incorporating clinical examination, photography, and imaging modalities [2]. Diverse grading systems evaluate bleb morphology based on clinical indicators, including vascularity, dimensions (height and width), microcystic changes, encapsulation, and delineation of diffuse versus demarcated zones, employing standardized clinical and photographic benchmarks [3-9].

In their research, Picht and Grehn identified specific characteristics associated with favorable outcomes in filtering blebs, such as a high prevalence of microcysts, minimal presence of conjunctival corkscrew vessels, and reduced height, as observed through slit-lamp examination [10]. Nonetheless, slit-lamp biomicroscopy presents limitations in directly visualizing the inner bleb structure and may require enhanced objectivity in assessment [11].

Additionally, ultrasound biomicroscopy (UBM) has been employed to evaluate bleb functionality. Studies have revealed that UBM's sensitivity is 91% in identifying functioning blebs and 70% specific in detecting non-functioning ones [12]. Given its reliance on a coupling fluid and direct contact, UBM encounters limitations as it may distort morphological features and pose infection risks, particularly in the early postoperative phase. In contrast, anterior-segment optical coherence tomography (AS-OCT) represents an advanced non-contact imaging modality capable of generating high-resolution cross-sectional images of the anterior segment of the eye. It uses the principle of the Michelson interferometer, which creates a reference beam of infrared light (1310 nanometer) against which it measures multiple other beams of light as they return from the variably reflective tissue layers of the eye. The detector detects the signals from the reference beam as well as the tissue layers of the eye. Multiple interference patterns are created for the signals received from the eye and the reference beam. The OCT images, thus formed, are based on analysis of interference patterns for echo time delay and intensity of reflected light. It offers meticulous evaluation of ocular surface and anterior chamber structures across diverse ocular pathologies without the concerns associated with contact-based techniques like UBM [13]. The bleb morphology can be studied using qualitative and quantitative AS-OCT parameters. Both parameters have been described in detail in the methodology.

Several studies have reported the morphology of bleb on AS-OCT taken at either two weeks or one month of follow-up (cross-sectional) and correlated with IOP at six months or one year of follow-up [14,15] or have studied them retrospectively [16,17]. Few studies have retrospectively correlated IOP six months post trabeculectomy with the serial AS-OCT imaging done postoperatively [18,19]. Hence, any bleb parameter can serve as a predictive factor for trabeculectomy failure and can be identified as a marker in decision-making during the weekly follow-up of trabeculectomy patients.

Objectives of the study

Our study aims to evaluate the bleb morphology in the early postoperative period and correlate the various bleb-related parameters on AS-OCT with intraocular pressure at day 1, day 7, days 21, and six weeks. Our study differs by assessing the bleb parameters of AS-OCT, including the success of trabeculectomy in both primary and secondary glaucomas and any bleb parameter that can be considered a predictive factor in trabeculectomy failure.

## Materials and methods

This research was carried out in the Department of Ophthalmology, Shri Chimanlal Harilal (CH) Nagri Municipal Eye Hospital, Ahmedabad. The Institutional Review Board, Smt. Nathiba Hargovandas Lakhmichand (NHL) Municipal Medical College, Ahmedabad reviewed and approved the study on December 8, 2022 (NHLIRB/2022/DECEMBER/8/NO.1). The study period for recruitment of cases was from January 1, 2023, to June 30, 2023. All the cases were followed up till six months after trabeculectomy. All the norms of the Declaration of Helsinki were accurately followed. The procedure details were explained to the patients, and written consent was obtained.

The sample size (n) was calculated using the formula n={2(Zα​)^2 ^X C X SD^2​^}/d^2 ^(where SD = anticipated standard deviation from the previous studies = 10, Zα​ = value of Z when a = 5% = 1.96, d = minimum expected mean difference = 4). So, the minimum sample size was calculated to be 50. The purposive sampling method was used in this study as the research had very specific research objectives. All the subjects diagnosed with primary or secondary open angle or angle closure glaucoma, on maximal medical therapy or intolerant to it, and who underwent trabeculectomy with or without mitomycin C (0.2 mg/ml for one minute) at our center were eligible for inclusion. Those patients who had undergone a previous trabeculectomy, a recent cyclodiode procedure, recent cyclocryotherapy, previous shunt surgery, pterygium surgery, combined phaco-trabeculectomy, small incision cataract surgery, recent phacoemulsification surgery, and those who refused to consent to the study were excluded. Preoperatively, 42 eyes were phakic (with natural lenses), and 13 eyes were pseudophakic (with artificial lenses).

The baseline intraocular pressure was the average IOP of a minimum of two measurements taken on two separate visits at different times of the same day. A single trained surgeon performed all the surgeries to remove the inter-operator bias.

The patients were followed up weekly for six weeks, at three months, and six months postoperatively for clinical evaluation and IOP measurement. At each follow-up, IOP was measured using a Goldmann applanation tonometer. As part of the study design, patients were called at any time of the day every week to ensure that any major diurnal fluctuations in IOP could be detected. All the AS-OCT imaging was done on day one, one week, three weeks, and six weeks postoperatively. Postoperative recovery was largely uneventful, with no major complications. However, during follow-up, three cases of bleb leak were noted in the third week, followed by two cases of bleb scarring and failure in the sixth week. Interventions were implemented as needed at each point, and details are provided in the results section.

AS-OCT imaging (Cirrus 3000, Carl Zeiss, Germany) utilized a standardized protocol to capture images of all blebs. Patients were instructed to gaze downward while the upper eyelid was delicately lifted to optimize bleb exposure without applying pressure to the globe or the bleb itself. Two radial and tangential scans were taken through the maximum elevation point on the bleb. The mean of the two measurements was used for analysis. A single skilled observer blinded to the clinical data observed AS-OCT images for any changes in morphologic features and noted the measurements.

Bleb parameters are classified as qualitative and quantitative. Bleb wall reflectivity (BWR) is a qualitative parameter categorized into uniform or multiform reflectivity depending upon the presence or absence of hyperreflective areas in the bleb wall.

Uniform reflectivity is characterized by a smooth, hyperreflective wall that lacks fluid-filled hypo-reflective spaces in the subconjunctival area. Conversely, multiform reflective walls exhibit small, fluid-filled spaces, appearing as hypo-reflective regions in either the conjunctiva or the bleb wall. Further classification of multiform bleb wall reflectivity has been done, as outlined by Khamar et al., into three subtypes: multiple internal layers, subconjunctival separation, and microcystic pattern [20]. Multiple internal layers manifest as hypo-reflective spaces in deeper conjunctival layers, resembling fluid channels parallel to the scleral surface. At the same time, subconjunctival separation presents numerous small fluid-filled spaces in superficial conjunctival layers. Microcystic patterns denote multiple rounded cystic areas in deeper layers, characterized by hypo-reflectivity. These patterns may appear independently or coalesce in various combinations.

**Figure 1:**
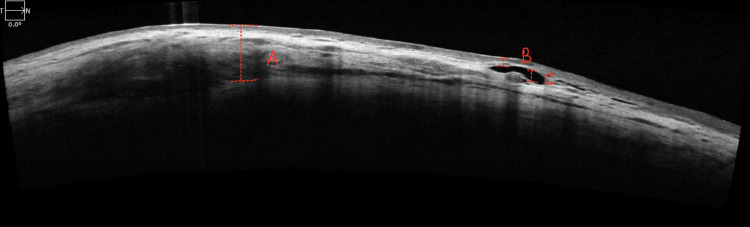
Shows a multifocal reflective bleb with different quantitative bleb parameters. A: Total bleb height; B: Bleb wall thickness, C: Fluid-filled cavity height.

Quantitative bleb parameters are described as total bleb height (TBH), bleb wall thickness (BWT), and fluid-filled cavity height (FFCH). The marking A in Figure 1 represents the total bleb height, which is the utmost vertical distance spanning from the conjunctival surface to the surface of the scleral flap. In cases where blebs exhibit uniform reflectivity, we solely measured the total bleb height. The marking B in Figure 1 shows bleb wall thickness, which refers to the distance from the initial reflective signal emanating from the conjunctiva to the uppermost extent of the subconjunctival fluid space. Given the potential variability in bleb wall thickness across the scan, measurements were confined to the minimum distance observed along the scan. The fluid-filled cavity height, depicted as C in Figure 1, denotes the maximal vertical extent of the area characterized by markedly reduced reflectivity, containing fluid and adjacent to the scleral flap.

Bleb function was a complete success if IOP was < or = 21 mm Hg without glaucoma medication, qualified success if IOP was < or = 21 mm Hg with glaucoma medication, and a failure if IOP was >21 mm Hg or < 6 mm Hg with or without glaucoma medications at the end of six months as per the world glaucoma association consensus [21].

Statistical analysis

All the data were entered into Microsoft Excel (Microsoft Corp., Redmond, WA) and analyzed using IBM SPSS Statistics for Windows, Version 22.0 (IBM Corp., Armonk, NY). All continuous variables are expressed as mean with standard deviation. Group differences between successful and failed groups were assessed using Spearman's correlation and Fisher's exact test. Correlation of IOP (at each follow-up and at six months) with bleb parameters was done using Spearman's correlation. Survival analysis was performed using the change of bleb wall reflectivity as the censoring variable, and Kaplan-Meier curves were plotted to depict the cumulative probability of change of bleb wall reflectivity from multiform to uniform at different point times post trabeculectomy. The receiver operator curve (ROC) was used to analyze the correlation between qualitative and quantitative parameters.

## Results

We included data from 55 eyes of 55 patients who completed a six-month follow-up post-trabeculectomy. The mean age of patients was 52.38 ± 14 years, of which 39 (70.9 %) were males. The preoperative mean visual acuity in the logarithm of the minimum angle of resolution (LogMAR) was 0.79 ± 0.55 of the study population. The mean preoperative IOP was 28.7 ± 9.9 mm Hg and 42.2 ± 9.9 mm Hg in successful and failed groups, respectively (p<0.001). The mean number of preoperative IOP-lowering drugs used was 4.6 ± 0.8 mm Hg. Forty-four eyes had primary glaucoma, and 11 eyes had secondary glaucoma (Table 1). Thirty-three subjects had early visual field (VF) damage, six had moderate damage, and 16 had advanced damage based on Humphrey Mean Deviation (dB). Table 2 categorizes participants based on the specific underlying causes of their glaucoma.

**Table 1:** Comparison of study participants based on outcomes. IOP: Intraocular pressure, LogMAR: Logarithm of the minimum angle of resolution, N/A: Not applicable. ^#^Spearman's correlation, ^$^Fisher's exact test, ^*^Significant.

Patients	Classification	Total	Successful group	Failed group	p-value
Number of eyes n (%)	N/A	55	45 (81.8%)	10 (18.2%)	N/A
Age in years (mean ± SD)^# ^	N/A	52.38 ± 14	53.16 ± 13.2	48.9 ± 17.9	0.45
Male: Female^$^	N/A	39:16	33:12	6:4	0.453
Preoperative IOP (mm Hg) (mean ± SD)^#^	N/A	31.16 ± 11.1	28.7 ± 9.9	42.2 ± 9.9	<0.001^*^
Preoperative visual acuity (LogMAR) (mean ± SD)^#^	N/A	0.79 ± 0.55	0.8 ± 0.5	0.7 ± 0.7	0.64
Glaucoma (Primary: Secondary)^$^	N/A	44:11	40:5	4:6	0.002^*^
No. of drugs used preoperatively n (mean ± SD)	N/A	4.6 ± 0.8	4.6 ± 0.8	4.7 ± 0.7	0.7
Visual field damage^$^, classified according to Humphrey Mean Deviation (dB)	Early <=6dB n (%)	33 (60%)	27 (60%)	6 (60%)	0.53
	Moderate >6dB -<12dB n (%)	6 (11%)	4 (9%)	2 (20%)	
	Advanced >= 12 dB n (%)	16 (29%)	14 (31%)	2 (20%)	

**Table 2:** Distribution of study participants according to cause of glaucoma (percentage). POAG: Primary open-angle glaucoma, PACG: Primary angle closure glaucoma, JOAG: Juvenile open-angle glaucoma, SO-induced glaucoma: Silicone oil-induced glaucoma, ICE syndrome: Iridocorneal endothelial syndrome, NVG: Neovascular glaucoma.

Diagnosis	N (%)	Successful group	Failed group
POAG	21 (38.18%)	20 (95.2%)	1 (4.8%)
PACG	20 (36.36%)	20 (100%)	0 (0.0%)
JOAG	3 (5.45%)	0 (0.0%)	3 (100%)
SO-induced glaucoma	2 (3.64%)	0 (0.0%)	2 (100%)
Traumatic glaucoma	3 (5.45%)	1 (33.3%)	2 (66.7%)
Steroid-induced glaucoma	2 (3.64%)	2 (100%)	0 (0.0%)
ICE syndrome	1 (1.82%)	0 (0.0%)	1 (100%)
NVG	2 (3.64%)	1 (50%)	1 (50%)
Pigmentary glaucoma	1 (1.82%)	1 (100%)	0 (0.0%)

We saw complete success in 30 eyes while qualified success in 15 at six months. Ten eyes had a complete failure of trabeculectomy at six months. Trabeculectomy was successful in 40 eyes with primary glaucoma and five with secondary glaucoma (p<0.002). Surgical intervention after trabeculectomy at the third week of follow-up was required in three cases: two out of the three patients needed bleb resuturing (bleb resuturing is indicated in bleb leak, bleb dysesthesia, bleb failure, encapsulated bleb, and infected bleb), and one underwent anterior chamber reformation with air. In the sixth week of follow-up, one case required bleb needling, and one had to be planned for Ahmed Glaucoma Valve (AGV). The latter had to be excluded from data analysis at the sixth-month follow-up.

On correlating various quantitative bleb parameters with IOP at different time points on follow-up (Table 3), there was a significant negative correlation between day 1 IOP and total bleb height (TBH). However, there was no significant correlation with IOP for the other time points (week 1, week 3, and week 6) and variables (bleb wall thickness and fluid-filled cavity height). At week 3 and week 6, the success rate of multiform blebs was significantly higher than blebs with uniform reflectivity (p-value = 0.001, p-value = 0.02). Among the multiform blebs, microcystic multifocal blebs were successful compared to other types at most time points. At week 3, the success rate of microcystic blebs was significantly higher than that of different types (p-value = 0.002).

**Table 3:** Correlation of IOP and bleb parameters after trabeculectomy at different time points on follow-up. IOP: Intraocular pressure. ^*^Significant.

Spearman's correlation		Day 1 IOP	Week 1 IOP	Week 3 IOP	Week 6 IOP
Total bleb height	Correlation coefficient	-.309	0.086	0.002	0.157
	p-value	0.022^*^	0.533	0.991	0.253
	N (number of patients)	55	55	55	55
Bleb wall thickness	Correlation coefficient	-0.023	0.097	0.024	0.057
	p-value	0.868	0.495	0.872	0.702
	N (number of patients)	53	52	48	47
Fluid-filled cavity height	Correlation coefficient	-0.154	0.045	-0.031	-0.001
	p-value	0.271	0.752	0.833	0.996
	N (number of patients)	53	52	48	47
Outcome		Success	Success	Success	Success
Multiform type	Cystic n (%)	30 (57.7%)	29 (54.7%)	22 (55%)	22 (53.7%)
	Cystic + subconjunctival separation n (%)	11 (21.2%)	12 (22.6%)	10 (25%)	8 (53.7%)
	Cystic + multiple internal layers n (%)	9 (17.3%)	9 (17%)	7 (17.5%)	8 (19.5%)
	p-value	0.65	1	0.002^*^	0.06
Bleb wall reflectivity	Uniform n (%)	2 (3.8%)	3 (5.7%)	1 (2.5%)	3 (7.3%)
	Multiform n (%)	50 (96.2%)	50 (94.3%)	39 (97.5%)	38 (92.7%)
	p-value	1	1	0.001^*^	0.02^*^

Correlation of various bleb parameters at each follow-up with IOP at six months (Table 4) showed that bleb wall reflectivity at week 3 and week 6 show a statistically significant correlation with IOP at six months (p<0.002). It also showed that none of the p-values for the quantitative bleb wall parameter correlation with IOP were statistically significant. Based on the binary logistic regression, none of the independent variables (Day 1 TBH, Day 1 BWT, Day 1 FFCH, Week 1 TBH, Week 1 BWT, Week 1 FFCH, Week 3 TBH, Week 3 BWT, Week 3 FFCH, Week 6 TBH, Week 6 BWT, Week 6 FFCH) were statistically significant predictors of the outcome variable. The model did not have strong predictive power for the binary outcome.

**Table 4:** Spearman's correlation of IOP and bleb parameters at different follow-ups with outcome at six months of trabeculectomy. IOP: Intraocular pressure. ^*^Significant.

Spearman's correlation		Day 1	Week 1	Week 3	Week 6
Intraocular pressure	Correlation coefficient	-0.005	-0.321	-0.636	-0.586
	p-value	0.972	0.018^*^	<0.001^*^	<0.001^*^
	N (number of patients)	54	54	54	54
Total bleb height	Correlation coefficient	0.059	-0.027	-0.013	-0.116
	p-value	0.672	0.846	0.927	0.402
	N (number of patients)	54	54	54	54
Bleb wall thickness	Correlation coefficient	0.021	-0.082	-0.234	-0.26
	p-value	0.881	0.565	0.113	0.078
	N (number of patients)	52	51	47	47
Fluid-filled cavity height	Correlation coefficient	0.064	0.011	0.176	0.206
	p-value	0.653	0.939	0.238	0.165
	N (number of patients)	52	51	47	47
Bleb wall reflectivity	Correlation coefficient	0.175	0.108	0.419	0.419
	p-value	0.205	0.435	0.002^*^	0.002^*^
	N (number of patients)	54	54	54	54

The consistency of bleb wall reflectivity showed a statistically significant association with the success of trabeculectomy (Table 5). Blebs that had no change in bleb wall reflectivity from day one to week six of follow-up were more likely to be successful at six months of follow-up (p-value ~ 0.029).

**Table 5:** Association of change in bleb wall reflectivity from day 1 to week 6 follow-up with outcome. ^#^Fisher's exact test.

Bleb wall reflectivity	Success	Fail	p-value
Same	41 (87.2%)	6 (12.8%)	0.029^#^
Change	4 (50%)	4 (50%)	

**Figure 2:**
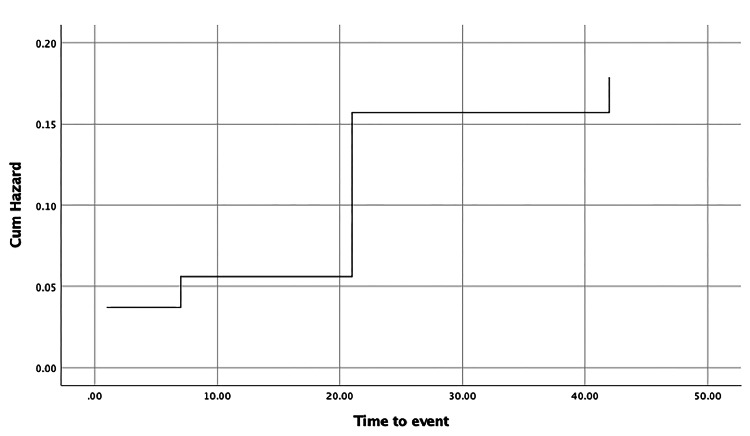
Kaplan-Meier curve showing the cumulative overall survival of change of bleb wall reflectivity from multiform to uniform at different time points post trabeculectomy. Cum hazard: Cumulative hazard.

**Figure 3:**
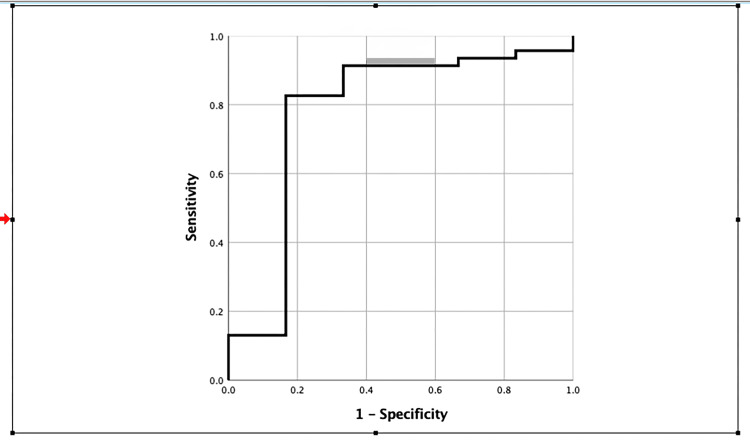
Receiver operator curve showing sensitivity and specificity of week 1 bleb wall thickness with the bleb wall reflectivity.

Figure 2 evaluated the survival probabilities of the bleb at specific time points: days 1, 7, 21, and 42 of follow-up. The cumulative hazard of change from multiform BWR to uniform BWR was estimated to be approximately 5.6%, 15.7%, and 17.9% at 7, 21, and 42-day follow-ups, respectively. The maximum change occurred between the day 7 and day 21 follow-up period, making it crucial to do a follow-up on the 21st-day post-trabeculectomy. When we correlated the qualitative bleb wall parameter (bleb wall reflectivity) with the quantitative parameters (Figure 3), it was found that bleb wall reflectivity remained the same on week 6 follow-up as on day 1, if week 1 BWT was less than 129.5 microns with 82.6% sensitivity and 83.3% specificity.

## Discussion

This prospective study observed the journey of maturing trabeculectomy blebs using AS-OCT bleb parameters in Indian eyes over six weeks. In this observational study of 55 eyes that underwent serial AS-OCT examination post trabeculectomy, multifocal bleb wall reflectivity showed a statistically significant association with intraocular pressure at six months. Higher preoperative IOP was associated with failure of trabeculectomy. Microcystic multifocal blebs were successful compared to other types at most time points. None of the quantitative bleb wall parameters correlated with IOP at six months of trabeculectomy. Our study additionally reported that BWR showed no change from day 1 to week 6 and had a successful outcome at six months. The maximum change in BWR was noted between the 7- and 21-day follow-up period, making it crucial to follow up on the 21st-day post-trabeculectomy. It was also found that BWR remained the same on week 6 follow-up as on day 1 if week 1 BWT was less than 129.5 microns with 82.6% sensitivity and 83.3% specificity.

Preoperative higher IOP was associated with trabeculectomy failure, as Tsutsumi-Kuroda et al. and Sugimoto et al. reported [14,22]. In a study by Khamar et al., where IOP at six months was correlated with morphology of blebs on AS-OCT done at one month postoperatively, multifocal bleb wall reflectivity was associated with an increased chance of success of trabeculectomy [20]. The odds of surgical success at six months of multiple layers combined with microcystic pattern was 35 times higher when the eyes had subconjunctival separation at one month, attaining a statistically significant difference (p<0.001). The results were like our study. Similar outcomes have been reported in other studies [16,19,23,24]. However, Güven Yilmaz et al. found that all biometric parameters (bleb base width, TBH, and BWT), except for bleb wall reflectivity, showed statistically significant differences between functional and nonfunctional blebs (p<0.05) [25].

We found none of the quantitative bleb wall parameters correlated with low IOP at six months of trabeculectomy. In a retrospective study by Tsutsumi et al., AS-OCT images of the bleb taken at two weeks post trabeculectomy were correlated with IOP at one year of follow-up, and they found that none of the bleb wall parameters (TBH, FFCH, BWT, and BWR) showed any correlation with IOP control [14]. Instead, the width of the filtration opening was a significant prognostic factor for the surgical success of trabeculectomy.

Hamanaka et al. found that IOP control was not statistically significant with bleb parameters in fornix-based trabeculectomy [26]. At the same time, limbal-based trabeculectomy showed that a combination of maximum TBH, minimum BWT, extent of bleb, and minimum BWT correlated with IOP control. They suggested that successful IOP control in fornix-based trabeculectomy may depend more on uveoscleral outflow.

Kokubun et al. retrospectively evaluated IOP at six months with bleb parameters measured at each postoperative follow-up of one week, two weeks, one month, three months, and six months [18]. The bleb parameters measured were cleft volume, bleb wall volume, vertical brightness of bleb wall, and horizontal brightness of bleb wall. The study design was very similar to ours; however, the measured parameters differed. They found the horizontal brightness of the bleb wall at two weeks to be the most substantial predictive factor for bleb status. The study's outcome by Tekin et al. disagreed with our research [27]. Higher AS‑OCT values for bleb height, wall thickness, and fluid‑filled cavity height were associated with more considerable functional success. They compared the AS-OCT values taken at six six-month follow-ups with IOP at the same visit.

Our study additionally reported that bleb wall reflectivity that showed no change from day 1 to week 6 had a successful outcome at six months. The maximum shift in bleb wall reflectivity was noted between the 7- and 21-day follow-up period, making it crucial to follow up on the 21st-day post-trabeculectomy. The six-week postoperative follow-up was done due to the analysis by Kaplan-Meier, which suggested that day 21 is a crucial day for follow-up. An AS-OCT of trabeculectomy bleb done on day 1 and at the third week postoperatively can indicate early bleb failure if the bleb wall reflectivity changes from multiform to uniform, even if IOP remains normal. Closer follow-up of these patients than the rest can be done with additional measures like bleb massage and subconjunctival injection of mitomycin C (MMC) or 5-fluorouracil (5-FU) for the survival of bleb.

Multiple studies have reported that a thicker bleb wall was associated with successful trabeculectomy. Waibel et al. conducted a similar study where BWT and FFCH were measured on day 1, week 1, weeks 2, 3, 4, and 12 and correlated with IOP [28]. AS-OCT assessments unveiled a noteworthy reduction in BWT within the encapsulating cohort compared to the functioning group, evident from the first week onward, persisting until week 12, notwithstanding any subsequent bleb needling intervention. Additionally, encapsulating blebs exhibited a progressive augmentation in bleb cavity height (BCH) relative to the functioning bleb cohort from week 2 through week 12, sustaining even post-bleb needling.

Our study shows no direct correlation between IOP and BWT. However, it shows that bleb wall reflectivity remained consistent if the thickness was less than 129.5 microns, with 82.6% sensitivity and 83.3% specificity. 

The probable reason for the failure of trabeculectomy in eyes with a thicker bleb wall is due to the reduced permeability and impaired filtration capacity of the bleb. Thicker bleb walls are often associated with increased fibrosis and scarring, which can decrease aqueous humor outflow [29]. This scarring process can obstruct the subconjunctival and sub-Tenon spaces, where fluid needs to diffuse, thereby leading to elevated IOP despite the presence of a filtering bleb. Moreover, the thickening of the bleb wall may be a result of an excessive wound-healing response, which includes the proliferation of fibroblasts and the deposition of extracellular matrix components [30]. These changes in the tissue structure compromise the bleb's functionality by creating a less porous barrier, making it more difficult for the aqueous humor to pass through effectively. Effective trabeculectomy depends on the creation of a functional filtering bleb with a thin, permeable wall that allows for adequate drainage of aqueous humor from the anterior chamber to the subconjunctival space [31]. When the bleb wall is thickened, this filtration process is disrupted, leading to trabeculectomy failure. Therefore, managing and mitigating postoperative fibrosis is crucial to improving the success rate of trabeculectomy in patients at risk of developing thicker bleb walls.

To the best of our knowledge and within our capacity for literature search, we have not found any similar study in the past by Indian authors.

Limitations of the study

Our study's relatively small sample size makes it challenging to interpret the relationship between bleb wall reflectivity and decreased thickness.

## Conclusions

Our study shows consistent multifocal bleb wall reflectivity from day 1 to week 6 of follow-up, which is more likely to have a successful trabeculectomy bleb at six months. Serial anterior-segment optical coherence tomography examination for changes in bleb wall reflectivity in early stages can be done to predict the fate of a bleb. The maximum shift in bleb wall reflectivity occurs between the first and third week of follow-up, making it a crucial period for follow-up to save these blebs.
